# Quantitative analysis of vascular signs on early postmortem multi-detector computed tomography

**DOI:** 10.1186/2193-1801-3-169

**Published:** 2014-04-01

**Authors:** Izumi Torimoto, Shigeo Takebayashi, Zenjiro Sekikawa, Noriko Nishimiya, Naoto Morimura, Tomio Inoue

**Affiliations:** Department of Radiology, Yokohama City University Graduate School of Medicine, 3-9 Fukuura, Kanazawa-ku, Yokohama, 236-0004 Japan; Department of Diagnostic Radiology, Yokohama City University Medical Center, 4-57, Urafune-cho, Minami-ku, Yokohama, 232-0024 Japan; Clinical Care and Emergency Center, Yokohama City University Medical Center, 4-57, Urafune-cho, Minami-ku, Yokohama, 232-0024 Japan

**Keywords:** MDCT, Postmortem imaging, Aorta, Cardiac arrest, Blood vessels

## Abstract

**Purpose:**

To clarify the postmortem multi-detector computed tomography (MDCT) vascular signs that occur shortly after death.

**Materials and methods:**

The vascular signs in MDCT images were evaluated quantitatively in 96 early postmortem cardiac arrest patients, 47 cardiac arrest patients who survived due to resuscitation and 47 control patients without cardiac arrest.

**Results:**

Elliptical (40 cases) or collapsed deformity (2 cases, in only the abdominal aorta) and high-attenuated sedimentation (19 cases in the aorta and 10 cases in superior or inferior vena cava) were limited to the postmortem patients. The incidence of elliptical deformity was higher for the abdominal aorta, descending thoracic aorta and ascending thoracic aorta in rank. The sedimentation was observed in the ascending thoracic aorta with a higher frequency than in the descending thoracic and abdominal aorta. A high-attenuating wall in any portion of the aorta was observed in 34 of the postmortem patients, 11 of the surviving patients and 10 of the control group, with a predominance of the ascending thoracic aorta.

**Conclusion:**

Elliptical deformity in the abdominal and descending thoracic aorta and high-attenuated sedimentation in the ascending thoracic aorta were shown to be signs of postmortem MDCT shortly after death.

## Introduction

It has been estimated that sudden cardiac arrest claims 100,000–120,000 per year in Japan and more than 400,000–500,000 lives per year in each the United States as well as Europe (Deasy et al. 2011). Survival until hospital discharge occurs in approximately 5% of out-of-hospital events and in 15% to 20% of in-hospital events in patients with cardiac arrest (Bobrow et al. 2010; Sandroni et al. 2007). In recent years, multi-detector computed tomography (MDCT), which provides increased spatial and temporal resolution, has been used in the post-resuscitation investigation in cardiac arrest patients if the spontaneous circulation is restored and the patient is sufficiently stabilized to be transferred to the CT room (Kim et al. 2012). Furthermore, MDCT can be used for postmortem imaging and has become a field of intensive study including quantitative evaluations (Roberts et al. 2012).

In Japan, postmortem MDCT instead of forensic autopsy is used to investigate the cause of death in out-of- hospital cardiac arrest patients without return of spontaneous circulation (ROSC) because the medical examiner system is not widely available (Shiotani et al. 2005).

Postmortem MDCT scanning often reveals high-attenuating aortic walls, collapse and hypostasis leading to a fluid-fluid level with high-attenuated sediment (Shiotani et al. [Bibr CR15][Bibr CR17]; Jackowski et al. 2006; Aghayev et al. 2006; Levy et al. 2007). Aghayev et al. (2006) performed quantitative analysis of the vascular system with postmortem MDCT. The imaging was performed at an average of 28 hours after death. To our knowledge, however, there have been few systematic and quantitative studies regarding MDCT imaging shortly after death in patients with cardiac arrest. It is important for forensic scientists and radiologists to know the time-dependent changes in a cadaver that begin immediately after death. The purpose of this study was to clarify which postmortem vascular signs occur shortly after death in cardiac arrest patients without ROSC.

## Material and methods

The institutional review board of Yokohama city university medical center approved this retrospective study. We confirmed that the legal representatives of the patients in this study were given a comprehensive written statement of information about the clinical study including MDCT and their consent was documented in the clinical records.

### Study group

The clinical and radiological database sets at our hospital were searched for 883 patients with cardiac arrest on arrival at the emergency center between April 2007 and December 2012. We extracted 285 patients in whom MDCT of the trunk was performed after cardiopulmonary resuscitation (CPR). Then, we excluded patients younger than 18 years (n = 18), patients with trauma (n = 72), aortic aneurysm with or without rupture (n = 34) and with intra-aortic balloon pumping (n = 13) and patients in whom over 10 minutes was required between stopping CPR and MDCT scanning (n = 5). Finally, the study group of cardiac arrest patients consisted of 143 patients, including 47 surviving patients (33%) and 96 early postmortem patients (67%). The study also included 47 control patients without cardiac arrest who underwent MDCT of the trunk and had a similar age distribution to the group of surviving patients.

The purpose of the postmortem MDCT was to investigate the cause of death. The surviving patients with ROSC who were sufficiently stabilized to be transferred to the MDCT suite in the emergency room, underwent post-resuscitation MDCT. The purpose of the post-resuscitation MDCT in the surviving patients was to rule out anoxic brain, retrosternal hematoma secondary to CPR, and non-cardiac causes of CPA such as pneumonia. In patients without ROSC, the determination of death was considered as the termination of CPR. The time between the termination of CPR and the performance of MDCT was 5 to10 minutes in both the early postmortem patients and the surviving patients.

### Treatment procedure

The CPR protocol in our institution was based on the guidelines of the American Heart Association in 2005 (Deasy et al. 2011). All of the patients with cardiac arrest received closed cardiac compression and positive pressure ventilation by paramedics. After they reached the emergency room (ER) at our hospital, our advanced CPR procedures were performed. A central venous line was inserted by the right subclavian approach and connected to acetated Ringer’s solution. Initially, epinephrine (1 mg) was administered intravenously every 3 minutes and CPR was continued for longer than 30 minutes in the patients without ROSC. However, the termination of CPR and the doses of epinephrine and the Ringer solution were determined by the ER physicians in charge.

### MDCT technique

All patients, including the control patients, underwent MDCT of the head and trunk without intravenous contrast enhancement using the MDCT system (Aquilion 16; Toshiba Medical Systems, Ohtawara, Japan). The trunk from the neck to the pelvis was scanned with 16 × 1-mm collimation (16 detectors with 1-mm section thickness), a beam pitch of 0.9375, a rotation speed of 0.5 seconds, a table speed of 15.0 mm per rotation and a tube current determined by automated tube modulation (120 kVp). Images were reconstructed at the MDCT console to a section thickness of 5 mm. Each image was sent to a picture archiving and communication system (PACS, Synapse ver. 3.1, Fuji-Film Medical Co., Tokyo, Japan).

### Clinical characteristics

After reviewing the clinical records of the 143 patients, one of the authors investigated the final clinical diagnosis, age, gender, duration of CPR, time between the termination of CPR and performing MDCT, the infused volume of Ringer solution and the infused dose of epinephrine. The body mass index was not available for the early postmortem patients because their height and weight were not recorded. Instead, we manually measured the thickness of the pectoralis major muscle at a 2-cm lateral to the sternum and the presternal subcutaneous fat depth on MDCT images.

### Measurements

The measurements of the greater vessels were performed by a board-certified radiologist with 10 years of experience in interpreting MDCT images and a postgraduate student with 3 years experience in CT image analysis. The two observers were also blinded to the patients’ histories and outcomes and the clinical data of the cases. The mean values of the measurements made by the two observers are described. All measurements were performed independently so that the inter-observer variability could be assessed. The ascending thoracic aorta, descending thoracic aorta and superior vena cava (SVC) on the axial image near the level of the pulmonary trunk were measured. Additionally, the abdominal aorta and inferior vena cava (IVC) on the image near the level of the renal arteries were measured.

**Figure 1:**
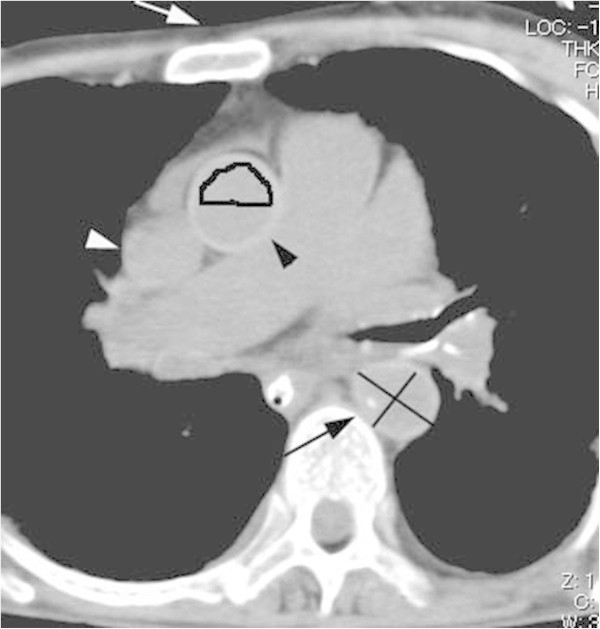
**An axial thoracic image from early postmortem MDCT in a 62-year-old man with cardiac arrest demonstrates the ascending thoracic aorta (black arrowhead) with a high-attenuation wall (70 HU) and a normal minor/major- axial diameter ratio (0.9).** An elliptical descending thoracic aorta (black arrow) with a minor/major-axial diameter ratio of 0.78 had a non-high attenuating wall measuring 58HU. No high-attenuated sedimentation was diagnosed in either great vessel. The presternal subcutaneous fat depth (white arrow) is 3.6 mm. Each line in the descending thoracic aorta indicates the minor- and major-axial diameter, respectively. The upper half of the lumen of the ascending thoracic aorta is manually traced for measuring the attenuation.

Both the minor-axial diameter and the major-axial diameter were measured in each portion of the aorta using the direct-line distance tool (Figure 1). To evaluate the configuration deformity, the ratio of the minor-axial diameter to the major-axial diameter was calculated in each portion of the aorta. The diagnosis of a collapsed vessel was made when the ratio was 0. According to a recent study measuring the ratio of the ascending thoracic, descending thoracic and abdominal aorta in ante-mortem patients, the mean values of the ratio were estimated at 0.86, 0.92 and 0.88, respectively (Takahashi et al. 2013). Then, we defined a ratio of 0.80 as the lower normal limit and a ratio of smaller than 0.80 as elliptical deformity. The observers measured the attenuation in the upper and lower halves of the lumens of the vessels (Figure 1). Attenuation in the wall of each portion of the aorta was measured in a magnified view on PACS; calcifications were avoided in these measurements. An area of the region-of-interest was manually placed for the measurements. The attenuation value was used when the region-of-interest was greater than 1.5 mm^2^, and the standard deviation was less than 10. A threshold of 65HU was used for a high-attenuating wall considering that the normal aortic lumen has 40–50 HU of attenuation: we used the threshold of 15HU as the discernible higher attenuation according to the threshold for enhancement on MDCT (Israel and Bosniak 2005). Similarly, the diagnosis of highly attenuated sedimentation was made when the attenuation in the lower half of the lumen was greater than 15 HU than that in the upper half (Israel and Bosniak 2005).

### Statistical analysis

We compared the mean values of the measurements and incidences of each vascular sign among the early postmortem, surviving and control patients. We also compared each variable among the clinical characteristics between the patients with each vascular sign and those without the sign in each patient group. The statistical analysis was performed using SPSS statistics (version 19.0, IBM. Co., Chicago, USA). The student’s *t* test was used to compare the continuous variables. Pearson’s chi-square test was used to compare the incidence of the investigated vascular signs and two categories of clinical characteristics. A *p*-value of 0.05 by a two-tailed test was considered statistically significant. For the inter-observer comparison, 100 patients were randomly selected. Inter-observer variability in the measurements was assessed with Bland-Altman analyses to estimate the confidence intervals for the bias and limits of agreement.

## Results

### Clinical characteristics

The clinical characteristics in the early postmortem, surviving and control groups are summarized in Table 1. There was no significant difference in the ratio of men to women among the groups. Furthermore, there was no significant difference in the mean values of age, the subcutaneous fat depth. or the muscle thickness. The mean values of the duration time of CPR, the infused volume of Ringer solution and the infused dose of epinephrine were greater in the early postmortem patients than the surviving patients (*p* < 0.0001 each). However, there was no significant difference in the time between the termination of CPR and performing MDCT between the early postmortem and surviving patients. The clinical diagnosis in each group is shown in Table 2. The diagnosis of arrhythmia or myocardial infarction was made in 37 (78.7%) of the surviving patients and the diagnosis was unknown in 54 (56.2%) early postmortem patients.

**Table 1:** **Clinical characteristics in early postmortem and surviving patients after cardiac arrest and control patients**

	Early postmortem patients (n = 96)	Surviving patients (n = 47)	Control patients (n = 47)	p values		
				Postmortem vs. Surviving	Postmortem vs Control	Surviving vs Control
Age (years)	70.1 ± 16.6 (18–94)	64.4 ± 16.5 (18–98)	65.7 ± 16.5 (18–96)	0.0730	0.1376	0.7843
Male/Female	62/34	30/17	26/21	0.9296	0.2848	0.4005
Pectoralis major muscle thickness (mm)	8.0 ± 2.8 (2–15)	8.3 ± 2.3 (3–13)	7.8 ± 1.9 (4–13)	0.5541	0.7346	0.3241
Presternal thickness (mm)	6.6 ± 4.1 (1–19)	5.7 ± 4.6 (1–30)	7.8 ± 3.9 (2–20)	0.2065	0.0985	0.4046
Duration time of CPR (minutes)	44.6 ± 9.1 (27–79)	18. 7 ± 8.8 (19–94)	NP	< 0.0001	NA	NA
Infused volume of Ringer’s solution(mL)	772.4 ± 359.2 (400–1980)	502.1 ± 153. 9 (300–1200)	NP	< 0.0001	NA	NA
Infused dose of epinephrine (mg)	4.5 ± 2.6 (0–9)	0.8 ± 1.1 (0–4)	NP	< 0.0001	NA	NA
Time between stopping CPR and MDCT scanning (minutes)	8.4 ± 1.2 (5–10)	8.5 ± 1.0 (5–10)	NP	0.4640	NA	NA

NP: not performed, NA: Not available.

NP: not performed.

**Table 2:** **Final diagnosis in early postmortem and surviving patients after cardiac arrest and control patients**

Clinical diagnosis	Early postmortem patients (n = 96) No. (%)	Surviving patients (n = 47) No. (%)	Control patients (n = 47) No. (%)
Arrhythmia/Myocardial infarction	0	37 (78.7)	7 (14.9)
Pneumonia	10 (10.4)	3 (6.4)	9 (19.1)
Cerebral vascular disease	12 (12.5)	0	2 (4.2)
Malignant neoplasm	5 (5.2)	3 (6.4)	10 (21.3)
Liver cirrhosis	5 (5.2)	1 (2.1)	6 (12.8)
Renal failure	2 (2.1)	3 (6.4)	10 (21.3)
Others	8 (8.3)	0	3 (6.4)
Unknown	54 (56.3)	0	0

### Measurement

**Figure 2:**
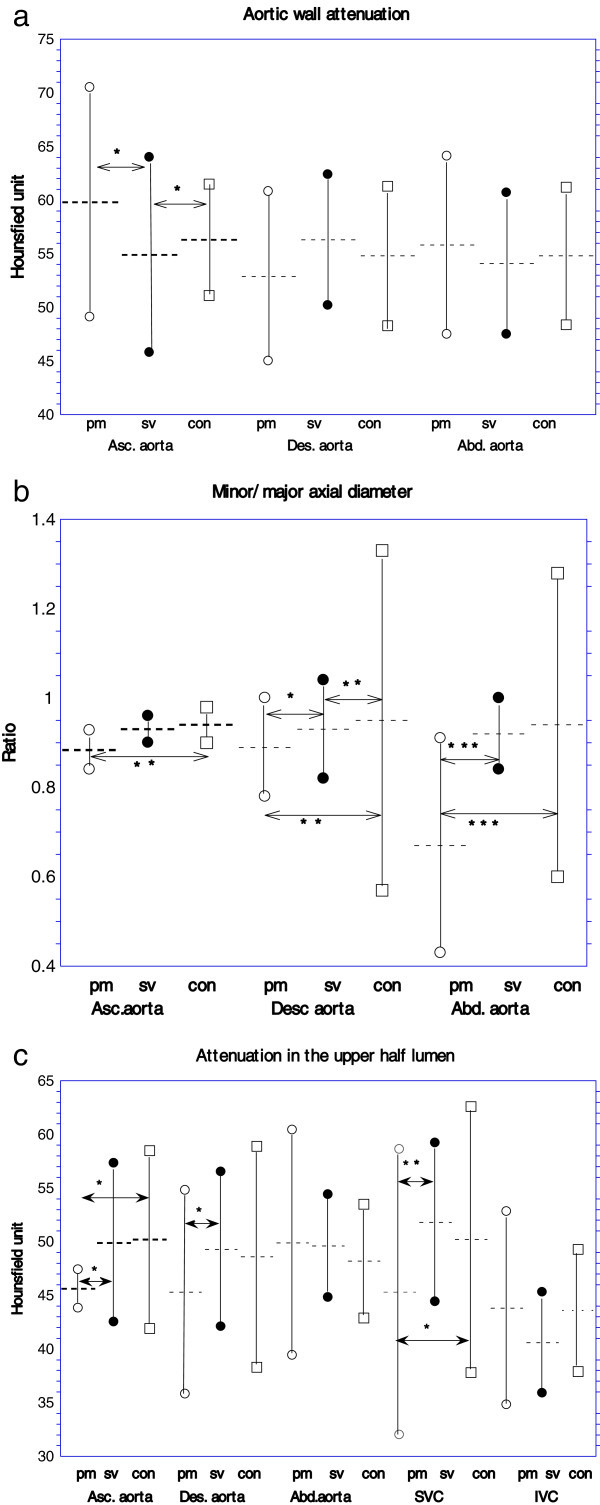
**Results of the measurements in the great vessels in early postmortem, surviving and control patients.** A dot line indicates a mean value and each upper and lower marker indicates a standard deviation. Arrow indicates a difference with a statistical significance in comparison of the mean value between the groups.(*;*p* < 0.05, **;*p* < 0.01,***; p < 0.001, ****;p < 0.0001). pm: early postmortem group, sv; survival group, con; control group. HU: Hounsfield unit **a**. Attenuation in the aortic wall. **b**. Ratio of minor axial diameter/major axial diameter of the aorta. **c**. Attenuation in the lower half lumen of the aorta, SVC and IVC.

Bland-Altman analysis demonstrated the agreement for the inter-observer variation between the two measurements of the diameter of the vessels (bias; -0.20 ± 8.93, 95% limit of agreement; -2.70 to 0.86, *p* = 0.154) and the measurements of attenuation of the vessels (bias; 0.89 ± 6.63, 95% limits of agreement; -0.37 to 2.15, *p* = 0.082). The early postmortem group had a significantly greater mean attenuation in the ascending aortic wall than the surviving and the control groups (Figure 2a). Each portion of the aorta in the postmortem group had a significantly smaller mean value for the minor/major-axial diameter ratio than that in the surviving and the control group (Figure 2b). With regard to the mean attenuation in the lumen, the postmortem group had a significantly lower value in the upper half of the lumen of the ascending thoracic aorta and the SVC than the other groups. Only the SVC had a significantly lower attenuation in the lower half of the lumen than the surviving patients (Figure 2c).

### Diagnosis of vascular signs

**Figure 3:**
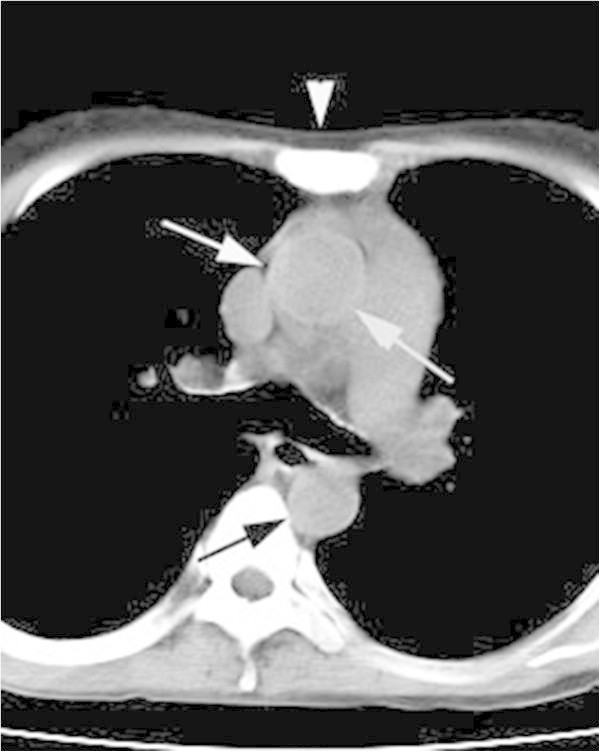
**An axial thoracic image from post-resuscitation MDCT in a 34 year-old man with cardiac arrest demonstrates high-attenuating walls of the ascending thoracic aorta (white arrows, 70 HU) and the descending thoracic aorta (black arrow, 59 HU).** No other vascular signs are demonstrated in the great vessels. Presternal subcutaneous fat layer (arrowhead) is 4-mm in thickness*.

**Figure 4:**
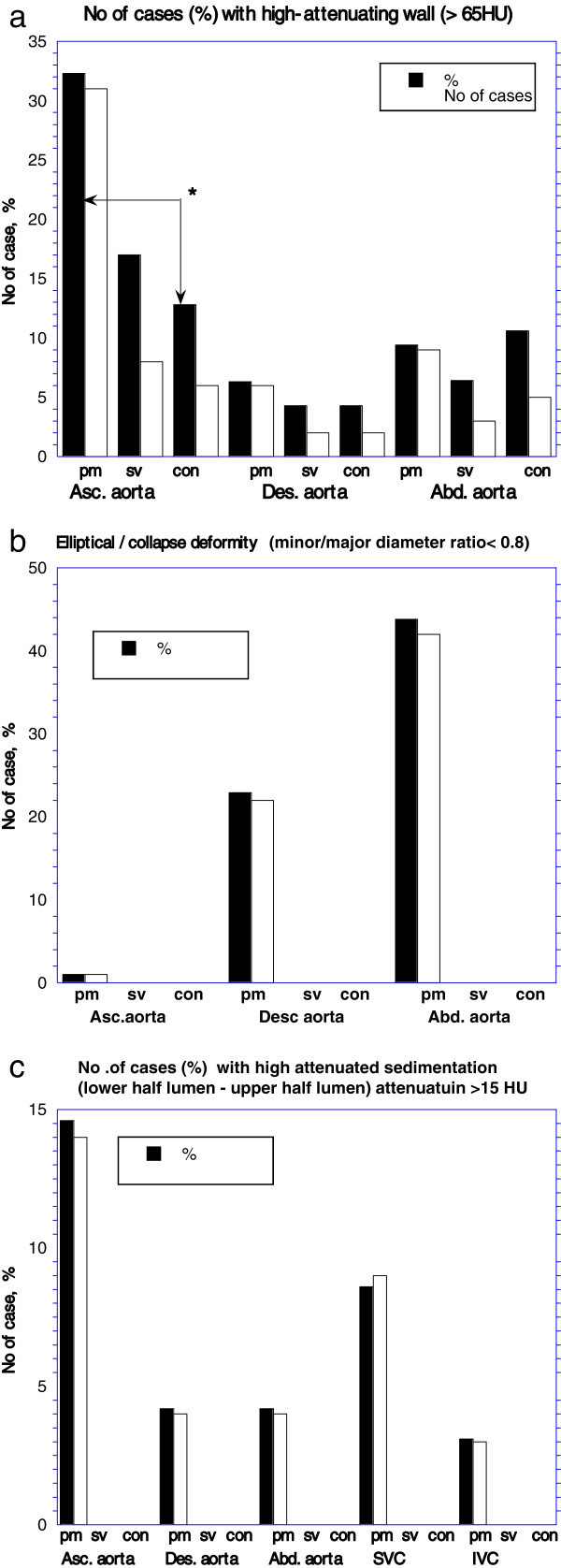
**Diagnosis of vascular signs based on quantitative analysis in early postmortem, surviving and control patients.** White bar indicates number of cases and black bar indicates percentage of cases. Arrow indicates a difference with a statistical significance in comparison of the mean value between the groups.(*;*p* < 0.05). pm: early postmortem group, sv; survival group, con; control group. HU: Hounsfield unit. **a**. High-attenuating wall of the aorta. **b**. Elliptical deformed or collapsed aorta. **c**. High-attenuated sedimentation.

**Figure 5:**
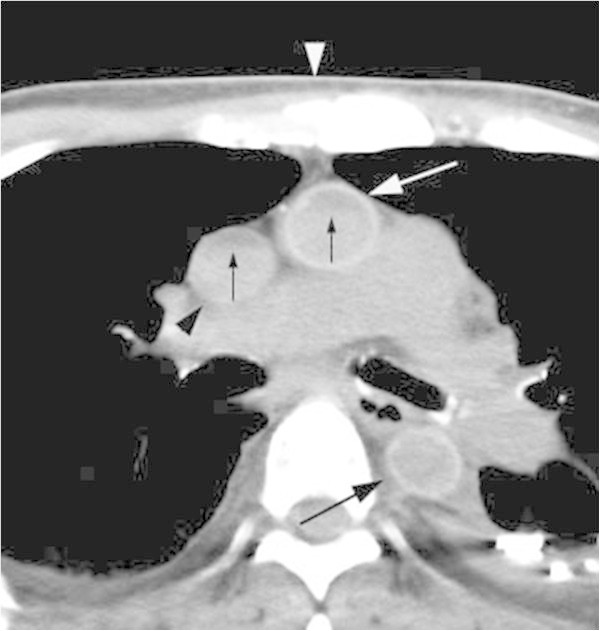
**An axial thoracic image from early postmortem MDCT in a 55 -year-old man with cardiac arrest demonstrates high-attenuating wall signs in each ascending thoracic aorta (large white arrow) and descending thoracic aorta (large black arrow) with the wall attenuation of 74 HU, 70HU, respectively.** Each ascending thoracic aorta and SVC has high-attenuated sedimentation with an attenuation difference of between the upper and lower halves of the lumen of 17 HU, 19 HU, respectively. Each descending thoracic aorta and abdominal aorta (not shown) are elliptical with minor/major-axial ratio of 0.76, 0.67, respectively. Presternal subcutaneous fat depth (white arrowhead) is 5-mm. A small black arrow in each ascending thoracic aorta and SVC indicates fluid-fluid level.

**Figure 6:**
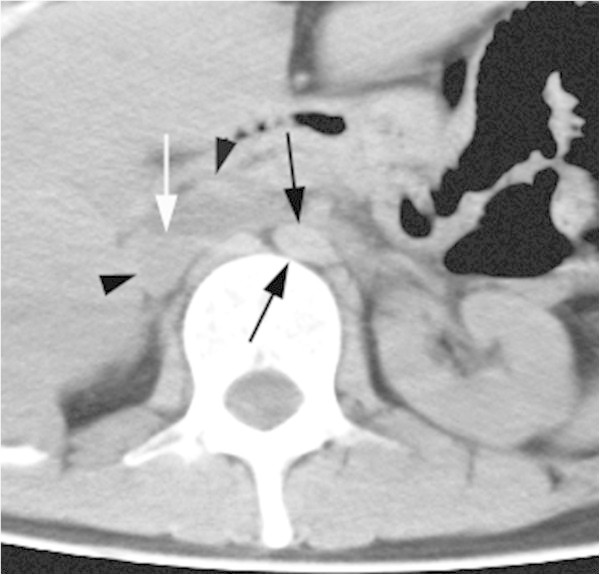
**An axial abdominal image from early postmortem MDCT in a 42 -year-old cardiac arrest woman demonstrates a small elliptical abdominal aorta (black arrows) with a minor/major-axial diameter ratio of 0.47.** The dilated IVC (black arrowheads) had high-attenuated sedimentation with a fluid-fluid level (white arrow) and attenuation difference of 22 HU between the upper and lower halves of the lumen.

The sign in the ascending thoracic aorta in the order frequency was the early postmortem group (32.3%), the surviving group (17.0%) and the control group (12.8%). A high attenuating wall was observed predominantly in the ascending thoracic aorta in each patients group (Figure 3). Significant differences were not observed in the incidences between the postmortem and surviving groups as well as the surviving and control groups. However, the differences between the postmortem and control patients were significant (*p* = 0.0123, Figure 4a). Thirty-four postmortem patients had the sign (Table 3, Figures 2, 5) [25, only ascending thoracic aorta; 6, each ascending thoracic, descending thoracic and abdominal aorta; 3, only abdominal aorta]. Collapsed aorta was observed in only the abdominal aorta in two early postmortem patients. Elliptical aorta was observed in the abdominal aorta (Figure 6), the descending thoracic aorta (Figures 1, 5) and the ascending thoracic aorta, in the order of decreasing frequency (Figure 4b) [22, both the descending thoracic aorta and abdominal aorta; 17, only abdominal aorta; 1, each ascending thoracic, descending thoracic and abdominal aorta].

**Table 3:** **Clinical characteristics of patients with or without high-attenuating aortic wall diagnosed based on quantitative analysis**

	High- attenuating aortic wall								
	Early postmortem			Surviving patients			Control patients		
Clinical characteristics	(+) 34 cases* (35.4%)	(-) 62 cases (64.6%)	p value	(+) 11 cases* (23.4%)	(-) 36 cases (76.6%)	p value	(+) 10 cases** (21.3%)	(-) 37cases (78.7%)	p value
Age (years)	70.0 ± 17.6 (25–94)	69.6 ± 17.1 (18–94)	0.9360	62.3 ± 15.2 (34–90)	65.5 ± 16.7 (18–98)	0.5680	69.3 ± 11.6 (48–88)	64.9 ± 17.4 (18–96)	0.4504
Male/Female	23/11	39/23	0.6421	6/5	23/13	0.5769	4/6	22/15	0.2721
Pectoralis major muscle thickness (mm)	7.8 ± 3.4 (2–15)	8.0 ± 2.3 (3–13)	0.8065	8.0 ± 2.0 (5–11)	8.4 ± 2.4 (3–13)	0.6465	8. 1 ± 1.8 (6–13)	7.7 ± 1.9 (4–13)	0.5385
Presternal fat depth mm)	4.3 ± 3.1 (1–17)	7.8 ± 4.1 (1–19)	<0.0001	4.0 ± 1.8 (1–7)	7.8 ± 6.0 (1–30)	0.0431	9.0 ± 4.1 (4–15)	7.4 ± 3.8 (2–20)	0.2501
Duration time of CPR (minutes)	46.6 ± 9.7 (30–72)	43.1 ± 9.0 (23–79)	0.8986	19.8 ± 8.1 (10–39)	18.4 ± 7.8 (5–38)	0.5673	NP		NA
Infused volume of the Ringer’s solution(mL)	779.7 ± 430.4 (420–1500)	759.7 ± 316.6 (400–1980)	0.1685	463.6 ± 143.4 (300–800)	505.4 ± 16.0 (300–1200)	0.4406	NP		NA
Infused dose of Epinephrine (mg)	5.0 ± 2.3 (0–9)	4.1 ± 2.8 (0–9)	0.7960	1.1 ± 1.2 (0–4)	0.8 ± 1.0 (0–4)	0.4098	NP		NA
Time between stopping CPR and MDCT scanning (minutes)	8.0 ± 1.2 (5–10)	8.3 ± 1.1 (5–10)	0.1138	8.5 ± 1.1 (7–10)	8.6 ± 0.9 (6–10)	0.6702	NP		NA

Values are the mean ± standard deviation. Parentheses indicate range. NP: Not performed.

NA: Not available, *Including 3 cases with high attenuating wall in both ascending thoracic aorta and abdominal aorta. **Including 4 cases with high attenuating wall in both ascending thoracic aorta and abdominal aorta.

High-attenuated sedimentation was diagnosed in 19 postmortem patients [14, the ascending thoracic aorta (Figure 5); 3, each ascending thoracic, descending thoracic and abdominal aorta; 1, both ascending thoracic and descending thoracic aorta; 1, abdominal aorta]. The ascending thoracic aorta had the sign with a higher frequency than in the descending thoracic and the abdominal aorta (Figure 4c). The sedimentation was also diagnosed in SVC (Figure 5) in 7 patients, both SVC and IVC (Figure 6) in 2 patients and only IVC in one patient.

### Vascular signs and clinical characteristics

Both the early postmortem and surviving patients with high-attenuating aortic wall had greater values of the mean subcutaneous fat depth than those without the sign (*p* < 0.0001, 0.0431, respectively). However, there was no significant difference in the mean depth between the control patients with and without a high-attenuating aortic wall (Table 3). The patients with an elliptical or collapsed aorta had a significantly lower mean age and received significantly greater mean dose of epinephrine than those without the deformed aorta (Table 4). There were no significant differences in the mean values of the clinical characteristics between early postmortem patients with high-attenuated sedimentation and those without the sedimentation.

**Table 4:** **Clinical characteristics, aortic deformity and high-attenuated sedimentation in early postmortem patients**

	Early postmortem patients (96 cases)								
	Elliptical/collapsed deformed			High-attenuated sedimentation					
	Aorta		p value	Aorta		p value	SVC/IVC		p value
Clinical characteristics	(+) 42 cases (43.7%)	(-) 54 cases (56.3%)		(+) 19 cases (19.8%)	(-) 77 cases (80.2.%)		(+) 10 cases (10.4%)	(-) 86 cases (89.6%)	
Age (Years)	65.4 ± 18.1 (18–94)	73.3 ± 14.8 (25–90)	0.0208	68.0 ± 16.7 (18–94)	70.2 ± 16.7 (28–94)	0.6337	68.8 ± 16.6 (18–94)	70.7 ± 17.1 (28–94)	0.6445
Male/Female	22/17	40/17	0.1661	12/7	50/27	0.8847	12/7	50/27	0.8847
Pectoralis major muscle thickness (mm)	7.9 ± 2.9 (2–15)	8.0 ± 2.7 (3–15)	0.8118	7.7 ± 1.7 (5–11)	8.0 ± 3.0 (2–15)	0.6916	8.2 ± 2.9 (3–11)	7.8 ± 2.6 (3–15)	0.6675
Presternal fat thickness (mm)	6.8 ± 4.3 (1–18)	6.4 ± 4.1 (1–19)	0.6217	7.5 ± 5.0 (1–18)	6.3 ± 3.9 (1–19)	0.2762	7.4 ± 5.2 (3–18)	6.5 ± 4.0 (1–19)	0.5294
Duration time of CPR (minutes)	46.4 ± 7.1 (33–60)	43.4 ± 10.1 (27–79)	0.1106	44.6 ± 9.5 (30–58)	44.7 ± 97.6 (27–79)	0.9735	44.0 ± 6.6 (33–60)	44.7 ± 9.6 (27–79)	0.7706
Infused volume of the Ringer’s solution (mL)	843.9 ± 431.0 (400–1500)	729.6 ± 297.6 (480–1980)	0.1598	717.6 ± 298.4 (400–1400)	776.0 ± 360.0 (400–1980)	0.5356	777.8 ± 365.5 (500–1700)	774.7 ± 361.1 (400–1980)	0.9740
Infused dose of epinephrine (mg)	5.4 ± 2.3 (0–9)	3.8 ± 2.8 (0–8)	0.0022	4.6 ± 2.6 (0–8)	4.4 ± 2.6 (0–9)	0.7900	4.6 ± 2.7 (0–8)	4.5 ± 2.6 (0–9)	0.8246
Time between CPR and MDCT (minutes)	8.1 ± 1.3 (5–10)	8.5 ± 1.1 (5–10)	0.0673	8.5 ± 0.9 (5–10)	8.3 ± 1.2 (7–10)	0.4047	8.6 ± 0.9 (7–10)	8.3 ± 1.2 (5–10)	0.3988

Values are the mean ± standard deviation. Parentheses indicate the range.

## Discussion

High-attenuating aortic walls, collapse and high-attenuated sedimentation are generally accepted as postmortem CT findings of the great vessels and heart (Shiotani et al. [Bibr CR15][Bibr CR17]; Jackowski et al. 2006; Aghayev et al. 2006; Levy et al. 2007). In most postmortem CT studies, the vascular signs were diagnosed based on imaging interpretation. However, a quantitative investigation of great vessels on postmortem MDCT revealed that a collapse of the descending thoracic aorta may be a reproducible radiologic sign of massive or fatal hemorrhage (Aghayev et al. 2006). In this study, we used the quantitative criteria for the vascular signs. The quantitative diagnosis is suggested to be superior to the diagnosis based on the imaging interpretation which is dependent on the abilities of the observers. There were no positive cases of an elliptical or collapsed deformity and high-attenuated sedimentation in the aorta of the surviving patients or the control patients.

The majority of the configuration deformities that occurred shortly after death were elliptical aorta, rather than collapse that is diagnosed when the two opposite walls of the vessels are touching each other or the anterior wall is concave (Jackowski et al. 2006). In this study, we defined the elliptical deformity that is similar to the definition of the ovoid- shaped deformity reported by Takahashi et al. (2013). Our observation that the deformity was predominantly shown in the abdominal aorta is accordance with their results. Takahashi et al. assumed that the predominance of the deformity was related to aortic elasticity which was greater in the ascending aorta and lower in the distal aorta. This elasticity maintains the round shape of the proximal aorta, and the loss of elasticity results in the deformations of the abdominal aorta shortly after death (Takahashi et al. 2013). According to the theory, an elliptical aorta occurs more frequently in elder patients because the elasticity of the aortic wall is decreased in elder patients. In this study, however, patients with a deformed aorta had a younger mean age than those without the deformed aorta. We suspect that the younger patients with the deformed aorta were under more catastrophic events because they received a higher dosage of epinephrine. In those patients, CPR is not effective to transmit pressure in the intra-thoracic aorta to the abdominal aorta.

We may also consider the effect of repeatedly administrated epinephrine on the aorta shortly after death because the terminal elimination half-life of epinephrine is approximately 8–13 minutes after intravenous administration (Gu et al. 1999). Epinephrine increases the CPR-generated aortic pressure via alpha-adrenergic–mediated vasoconstriction, which increases the coronary perfusion pressure by decreasing the blood flow to all other organs (Callaway 2012). In this case, the tone of the ascending thoracic aorta connected to the coronary arteries is preserved, but downstream aorta, such as the abdominal aorta, loss of tone with the reduced elasticity.

High-attenuated sedimentation in the ascending thoracic aorta was also shown to be signs shortly after death postmortem MDCT. A high-attenuated sedimentation, which is also called the fluid-fluid level or hematocrit effect, is thought to be hypostasis (Shiotani et al. [Bibr CR15][Bibr CR17]; Jackowski et al. 2006; Aghayev et al. 2006; Levy et al. 2007). In this study, early postmortem MDCT shortly after death revealed this sign in the aorta in approximately 20% of cases, and SVC or IVC was observed in 10% of cases with a predominance of the ascending thoracic aorta and SVC. The frequency of this phenomenon would increase with the passage of time (Takahashi et al. 2013). Delayed postmortem CT within two hours after death revealed this sign in 56% of cases (Shiotani et al. 2002a). Postmortem MDCT that was performed prior to autopsy revealed this sign in the great vessels and cardiac chambers in all investigated cases (Jackowski et al. 2006). A cessation of circulation induces hypostasis of the blood, which occurs earlier in the great vessels with large lumens and in the heart chambers. The vascular sign may indicate that both the clotting process and thrombolysis are occurring in parallel. Thrombolysis occurs due to an increase in plasminogen activator release by the endothelium, and the serum platelets and serum proteins are functionally intact for a variable time shortly after death (Weisfeldt and Chandra 1981). Lower attenuation in the upper lumen of larger vessels, such as the ascending thoracic aorta, the descending thoracic aorta and the SVC, is thought to be the result of thrombolysis.

A high-attenuating wall sign in the aorta is not a specific postmortem MDCT finding. In this study, the surviving patients and the control patients were positive for the sign with an approximate incidence of 21–23%, and 35% of the early postmortem patients had the sign. The high-attenuating wall in the control group is caused suggestively by atherosclerosis (Landay and Virolainen 1991). In postmortem patients, contraction of the aortic wall is thought to cause the high attenuation (Shiotani et al. 2002b). Intense sympathetic stimulation shortly after the cessation of circulation causes contraction of smooth muscles in the aortic wall which are intact for up to 6 hours following cardiac arrest (Guyton and Hall 2006). In this study, both early postmortem and surviving post-CPR patients with high-attenuating aortic wall and a predominance of the ascending thoracic aorta had a significantly smaller subcutaneous fat depth than those without the sign. This finding suggested that decreasing presternal subcutaneous fat depth may be associated with damage to the wall of the ascending thoracic aorta by a direct force causing sternal compressions during CPR. The damage is associated with microscopic hemorrhage and may be one of factors causing a high-attenuating wall in patients who received CPR. But increased subcutaneous fat depth is associated with significantly decreased injury severity and may be protective against injuries by cushioning (Wang et al. 2003).

To summarize, we offer the following conclusions from the series of early postmortem cardiac arrest patients, cardiac arrest patients who survived due to resuscitation and control live patients without cardiac arrest. Elliptical deformity in the abdominal and descending thoracic aorta and high-attenuated sedimentation in the ascending thoracic aorta were shown to be signs using quantitative analysis of postmortem MDCT shortly after death. However, our study has some limitations: First, in this retrospective study using quantitative evaluation of the measurements, the observer may have affected the measurements of the MDCT images. Second, our study did not evaluate antemortem MDCT images for a comparison with postmortem or post resuscitation MDCT in the same patients (Takahashi et al. 2013; Hyodoh et al. 2012), although we did compare the images with a group of control patients.
